# Research Advances in Plant Pyruvate Kinase

**DOI:** 10.3390/ijms27146346

**Published:** 2026-07-17

**Authors:** Ruixiao Peng, Fudeng Huang, Yong He, Junfeng Xu, Ying Zhu, Mengyun Ren, Yuanyuan Hao, Zhihong Tian

**Affiliations:** 1 Engineering Research Center of Ecology and Agricultural Use of Wetland, Ministry of Education, College of Life Science, Yangtze University, Jingzhou 434025, China; yuzhongprx@163.com (R.P.); warers@yangtzeu.edu.cn (Y.H.); 2 Institute of Crop and Nuclear Technology Utilization, Zhejiang Academy of Agricultural Sciences, Hangzhou 310021, China; pahfd@126.com (F.H.); renmengyun@zaas.ac.cn (M.R.); 3 State Key Laboratory for Quality and Safety of Agro-Products, Zhejiang Academy of Agricultural Sciences, Hangzhou 310021, China; njjfxu@163.com (J.X.); yzhuzaas@163.com (Y.Z.)

**Keywords:** pyruvate kinase, PKc, PKp, dynamic subcellular distribution, plant growth and development

## Abstract

Pyruvate kinase (PK) is the terminal rate-limiting enzyme of glycolysis and occupies a central position in plant energy metabolism and carbon skeleton allocation. Plant PK isoenzymes comprise the cytosolic pyruvate kinase (PKc) and the plastidic pyruvate kinase (PKp), which differ markedly in gene origin, protein structure, subcellular localization, and physiological function, exhibiting independent evolutionary histories and functional diversification. Recent studies have revealed that PKc possesses dynamic subcellular distribution, allowing it to shuttle among the cytosol, mitochondria, and nucleus, where it participates in stress responses and epigenetic regulation through protein–protein interactions. PKp is localized to plastids and connects carbon metabolism with lipid biosynthesis and the methylerythritol phosphate (MEP) pathway by supplying pyruvate, thereby playing critical roles in seed development and oil accumulation. This review comprehensively summarizes recent advances in plant PKc and PKp concerning protein structure and subunit composition, tissue-specific expression, subcellular localization, protein interaction networks, activity regulation, and their effects on plant growth, development, and stress responses. In addition, phylogenetic tree, motif, and domain analyses of pyruvate kinase genes from *Oryza sativa* (rice), *Glycine max* (soybean), *Gossypium hirsutum* (cotton), *Solanum tuberosum* (potato), *Arachis hypogaea* (peanut), and *Arabidopsis thaliana*, as well as promoter cis-element analyses, are performed. This review aims to provide theoretical references for crop quality improvement and stress-resilient breeding.

## 1. Introduction

**Figure 1:**
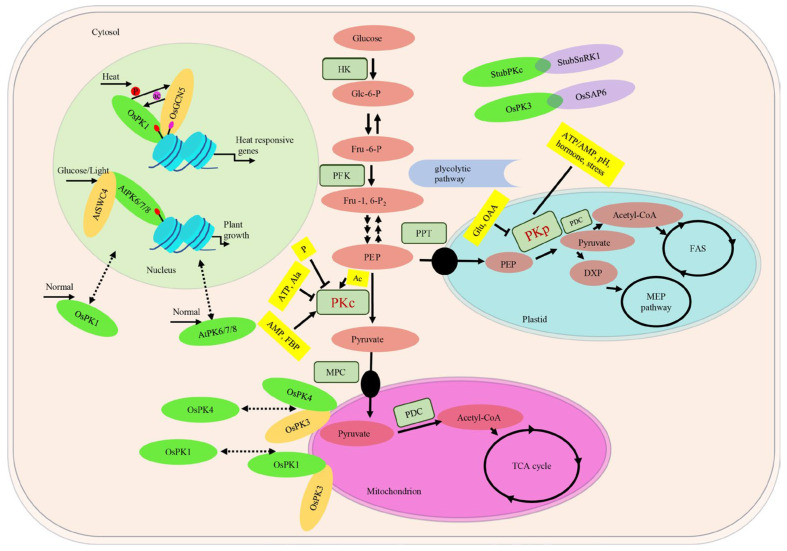
Metabolic pathways, subcellular localization, and protein interaction networks of cytosolic (PKc) and plastidic (PKp) pyruvate kinase in plant cells. Arrows indicate metabolic flux; ▸ denotes positive activation, ⊣ denotes negative inhibition, p denotes phosphorylation, and ac denotes acetylation, dashed line represents shuttling between the cytoplasm and other organelles, circular arrow symbols indicate the directionality of individual metabolic pathways, circle straddling the arrow represents inter-organellar transport.

Glycolysis is the central pathway of energy metabolism in living organisms and a central regulator connecting primary and secondary metabolism. In higher plants, the glycolytic pathway exhibits unique complexity and diversity—glycolysis in plant cells does not occur exclusively in the cytosol, but rather takes place simultaneously in both the cytosol and the plastid compartments [1]. This spatially compartmentalized metabolic organization endows plants with a highly flexible capacity for regulating carbon metabolism. This pathway comprises ten highly conserved, sequential enzymatic reaction steps, three of which are irreversible: glucose phosphorylation, fructose-1,6-bisphosphate formation, and pyruvate generation, catalyzed respectively by hexokinase (HK), phosphofructokinase (PFK), and pyruvate kinase (PK). These three steps constitute the key regulatory nodes governing glycolytic flux (Figure 1) [2]. Pyruvate, the central product of this pathway, serves as a pivotal hub in the plant cellular metabolic network. It can be transported into mitochondria via the mitochondrial pyruvate carrier (MPC) and converted to acetyl-CoA by the pyruvate dehydrogenase complex (PDC), entering the tricarboxylic acid (TCA) cycle for energy production [3,4]. Alternatively, pyruvate can be converted to acetyl-CoA by PDC (the gene product of the mitochondrial PDC homolog). within plastids, entering the fatty acid biosynthesis pathway, or transformed into 1-deoxy-D-xylulose-5-phosphate (DXP) to enter the methylerythritol phosphate (MEP) pathway, contributing to the biosynthesis of plant terpenoids [4,5] (Figure 1). Pyruvate may also regenerate glucose through gluconeogenesis, or be carboxylated by pyruvate carboxylase (PC) to form oxaloacetate (OAA) for participation in amino acid biosynthesis [5,6]. The multiple metabolic fates of pyruvate establish it as a critical node linking energy metabolism with biosynthetic processes [7]. Consequently, the supply level of pyruvate directly influences the energy status, biosynthetic capacity, and environmental adaptability of plants [5].

PK catalyzes the final rate-limiting step of glycolysis, converting phosphoenolpyruvate (PEP) and ADP into pyruvate and ATP. This reaction is thermodynamically irreversible (ΔG°′ ≈ −31.4 kJ/mol), positioning PK as a critical “valve” controlling the direction and rate of glycolytic flux [8]. In contrast to animals and microorganisms, plant PK exhibits a unique dual origin, giving rise to two independent PK isozyme systems: cytosolic pyruvate kinase (PKc) and plastidic pyruvate kinase (PKp). The sequence similarity between plant PKc and PKp is approximately 35–40%, which is lower than that between the plastidic enzyme and its homologs from other eukaryotes, suggesting that plant PKp may have undergone faster divergence during evolution [9]. PKc is homologous to the PKc of eukaryotes and originated from the nuclear genome of the eukaryotic host, whereas PKp is of prokaryotic origin, with its gene transferred to the plant nuclear genome from a cyanobacterial ancestor during endosymbiotic events [10,11,12]. This dual origin confers remarkable structural, subcellular, and functional diversity upon plant PK. Plant cells contain multiple subcellular compartments (cytoplasm, plastids, mitochondria, and nucleus), each with distinct pyruvate demands and regulatory mechanisms. Consequently, PK function extends well beyond energy production, actively participating in carbon flux partitioning and metabolic signal transduction [2,13].

**Figure 2:**
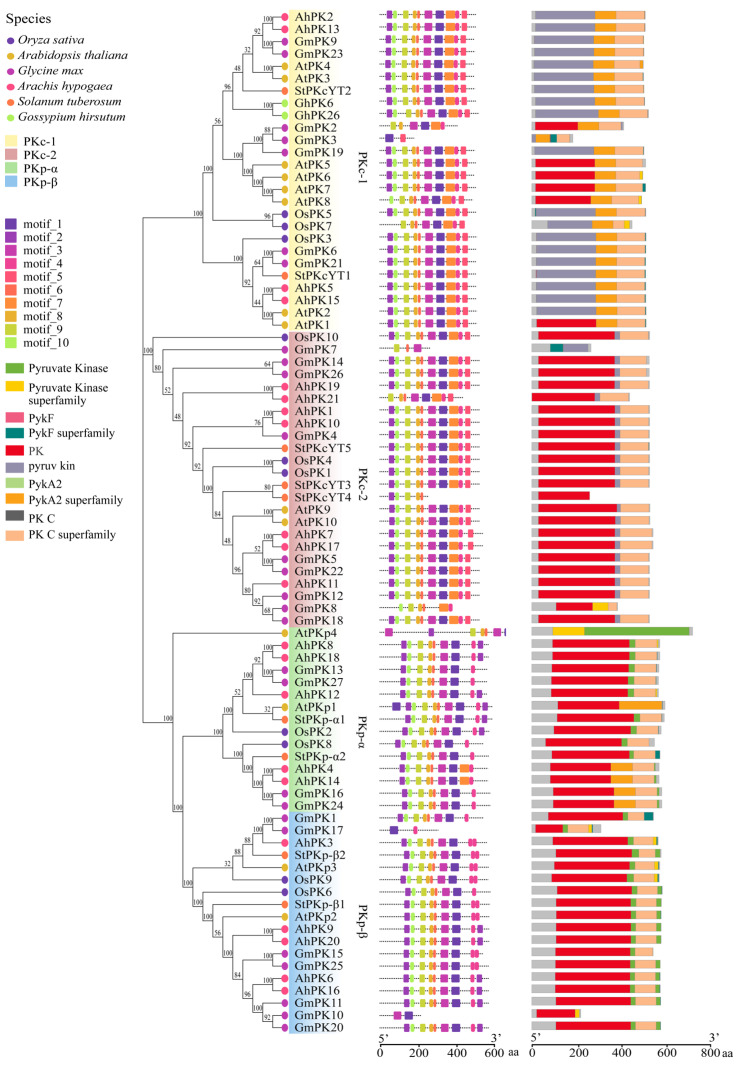
Phylogenetic relationships, conserved motifs, and domain analysis of PKs from six plant species (*Oryza sativa* (rice), *Glycine max* (soybean), *Gossypium hirsutum* (cotton), *Solanum tuberosum* (potato), *Arachis hypogaea* (peanut), and *Arabidopsis thaliana*). The phylogenetic tree was constructed using the maximum likelihood (ML) method with 1000 bootstrap replicates. A total of 10 conserved motifs were identified using the MEME (Multiple Em for Motif Elicitation) suite. The conserved domains of the PKs proteins were analyzed using NCBI-CDD. Visualization of the results from both analyses was performed using TBtools.

For a more comprehensive phylogenetic comparison of the PK family, two model plants (*Oryza sativa* (rice) and *Arabidopsis thaliana*) and four agriculturally and economically important crops (*Glycine max* (soybean), *Gossypium hirsutum* (cotton), *Solanum tuberosum* (potato), *Arachis hypogaea* (peanut)) were chosen for evolutionary tree analysis. These six species, which cover different product organ types—including oilseeds, fibers, and tubers—have all benefited from high-quality genome sequencing and annotation and have been the subject of functional studies on the PK family (Figure 2, Tables S1–S3). The tree is rooted and displays two well-supported major clades corresponding to PKc (shaded in yellow and red) and PKp (shaded in green and blue). The PKc clade further subdivides into two distinct subclades, designated PKc-1 and PKc-2, while the PKp clade is partitioned into PKp-α and PKp-β subclades, with all subtypes present in both monocots and eudicots. Species-specific gene duplications are evident: polyploid species (Gm, Ah, and Gh) have experienced significant PKc-1 and PKc-2 lineage expansions, retaining 7–10 and 4–7 members, respectively. In contrast, *O. sativa* and *A. thaliana* possess relatively compact PKc gene families, with 6 and 10 members total, respectively. Notably, the sequences of GmPK2/3 in PKc1, GmPK7/8 and StPKcYT4 in PKc2, AtPKp4 in PKp-α, and GmPK17/10 in PKp-β all differ markedly from their closely related members within their respective subclades. Ten motifs architecture is broadly conserved within each subclade but differs between PKc and PKp, consistent with their distinct evolutionary origins. PKc-1 and PKc-2 members generally carry the full complement of 10 motifs, whereas most PKp members lack one or more motifs in the C-terminal region, reflecting domain rearrangements associated with plastidial targeting and subunit diversification. As the protein domain architecture, both PKc and PKp proteins contain the conserved PK catalytic domain, which spans approximately 400–500 amino acid residues. In addition, several PKp members (particularly in the PKp-β subclade) contain bacterial-origin domains (PykF, PykA), consistent with the cyanobacterial endosymbiotic origin of the plastidial PK lineage [14]. The PKp proteins are generally shorter (~500–600 aa) than PKc proteins (~520–570 aa) due to the absence of extended regulatory domains. The PykF superfamily domain detected in GhPK and AhPK proteins suggests possible horizontal gene transfer events or distinct evolutionary trajectories in certain eudicot lineages.

**Figure 3:**
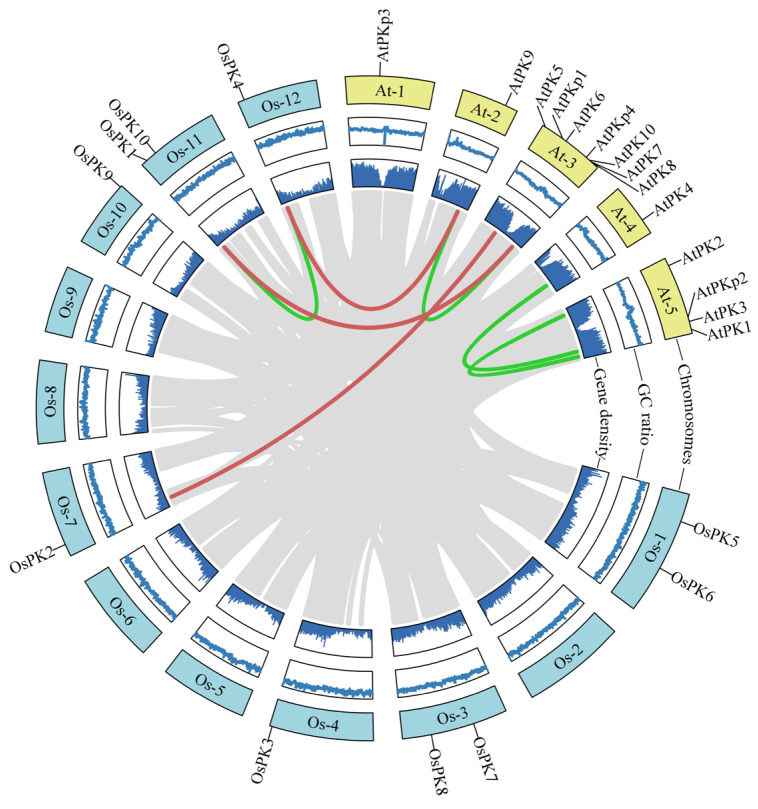
Collinearity analysis of the PK gene family between rice and *Arabidopsis*. Gray lines represent all homologous gene pairs between the two species, red lines represent orthologous PK genes, and green lines represent paralogous PK genes. The outermost ring represents chromosomal locations, the second ring represents GC content, and the innermost ring represents gene density.

Given that the genome sequences of Arabidopsis and rice are both well-annotated and comprehensively assembled, synteny analysis between these two species can serve as an ideal framework for inferring whether PK genes are evolutionarily conserved across monocots and dicots (Figure 3). The result showed that the common ancestor of monocots and eudicots possessed a highly limited number of ancestral PK gene copies. Intraspecific homology analysis revealed that the rice genome contains only one pair of PK paralogs (*OsPK1* and *OsPK4*), whereas *Arabidopsis* retains three pairs of PK paralogs (*AtPK1/AtPK2*, *AtPK3/AtPK4*, and *AtPK9/AtPK10*) (Figure 3).

**Figure 4:**
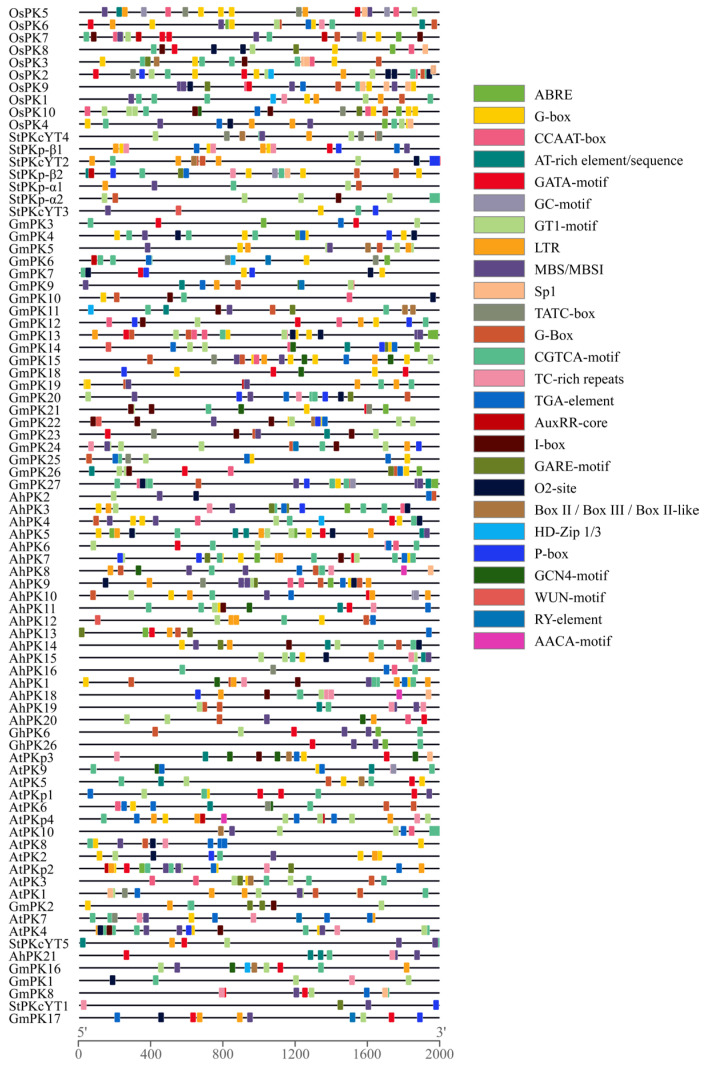
Positions of 25 important cis-acting elements in the promoters (2 kb upstream of ATG) of PKs genes from six plant species(*Oryza sativa* (rice), *Glycine max* (soybean), *Gossypium hirsutum* (cotton), *Solanum tuberosum* (potato), *Arachis hypogaea* (peanut), and *Arabidopsis thaliana*), with the analysis results generated by PlantCARE.

The cis-acting elements in the 2000 bp promoter regions upstream of all PK genes from these six plant species were also systematically analyzed, and identified a total of 25 elements, which were classified into four functional categories: core promoter/basal transcription, light responsiveness, phytohormone responsiveness, and biotic/abiotic stress responsiveness (Figure 4 and Figure S1, Tables S4 and S5). Among them, light-responsive elements such as G-box, GT1-motif, and I-box were widely distributed, with G-box being the most prevalent, suggesting that PK expression may be coordinately regulated with photoperiod [15]. ABRE elements were particularly abundant in peanut and soybean, while the presence of CGTCA-motif and GARE-motif indicated potential crosstalk between carbon metabolism and hormonal signaling pathways, including jasmonic acid and gibberellin [16]. Meanwhile, the identification of stress-related elements such as MBS/MBSI, LTR, and TC-rich repeats further supported the functional relevance of PK genes in stress responses [17]. Moreover, differences in the distribution patterns of cis-elements among species and paralogous genes suggested functional divergence and subfunctionalization of the PK family during evolution. These findings provide new insights into the transcriptional regulatory networks governing PK gene expression and highlight the potential central role of PK in integrating metabolic, hormonal, and environmental signals.

In recent years, with the completion of multiple plant genome sequencing projects and the widespread application of functional genomics technologies, significant progress has been made in the systematic identification and functional characterization of plant PK gene families [18,19,20,21,22,23,24,25,26,27]. In this review, using PKc and PKp as the two principal subtypes, we systematically summarize the research advances in plant pyruvate kinase across six dimensions: protein structure and subunit composition, tissue-specific expression, subcellular localization, protein–protein interaction regulatory networks, activity regulation mechanisms, and effects on plant growth and development (Table 1).

### 1.1. Cytosolic Pyruvate Kinase (PKc)

#### 1.1.1. Protein Size and Subunit Composition

Plant PKc genes typically contain 10–12 exons and encode proteins with a molecular weight of approximately 55–60 kDa (500–530 amino acid residues) [2,13]. In higher plants, PKc assembly tends toward heteromerization to accommodate more complex regulatory functions. PKc from developing endosperm of castor bean (*Ricinus communis*) and tobacco (*Nicotiana tabacum*) leaves exists primarily as a heterotetramer, composed of two subunits with similar molecular weights (~57 kDa and ~56 kDa) but distinct biochemical properties [3]. PKc from ripe banana (*Musa acuminata*) fruit forms a homotetramer of approximately 240 kDa, with a subunit size of approximately 57 kDa [6]. PKc from potato tubers was identified as a heterodimer, and this relatively simple assembly pattern is adapted to its specific metabolic function in storage organs [19]. In lower plants, PKc tends to form homomultimers. PKc from the green alga *Selenastrum minutum* was confirmed as a homotetramer composed of a single 57 kDa subunit [20].

PKc genes exist as multigene families in plants, with significant variation in gene copy number among species. The *Arabidopsis thaliana* genome encodes 10 PKc genes (*AtPK1–AtPK10*), while 6 *OsPK* genes have been identified in rice (*Oryza sativa*) [18]. In soybean, 27 *GmPK* genes were identified, of which 16 belong to the PKc subfamily [21]; in peanut, 11 *AhPKc* genes were identified (4 *PKc-1* and 7 *PKc-2*) [22]; in *litchi* (*Litchi chinensis*), 19 *LcPK* genes were identified, distributed unevenly across 15 chromosomes [23]; in upland cotton, 19 PKc genes were identified, with 8 pairs exhibiting homologous relationships, closely associated with its allopolyploid genome origin [24,25]; in tiger nut (*Cyperus esculentus*), 4 PKc genes (*CePKc1–CePKc4*) were identified [26].

Regarding sequence characteristics, plant PKc proteins all contain a highly conserved PK catalytic domain, including the PEP-binding domain, metal ion binding sites (K^+^ and Mg^2+^), and allosteric regulatory sites [2]. *Arabidopsis* AtPKc1 and AtPKc2 share over 50% sequence similarity with mammalian muscle-type PK (M1-PK) and liver-type PK (L-PK), indicating high conservation of PKc genes during eukaryotic evolution [2,13].

#### 1.1.2. Tissue- and Organ-Specific Expression Profiles

PKc exhibits highly differentiated expression patterns across various plant tissues and organs, and this differential expression forms the molecular basis for specific metabolic functions of different subtypes. In *Arabidopsis*, systematic analysis of the transcriptional characteristics of 10 *AtPKc* genes revealed significant differences in expression patterns among subtypes. *AtPK6*, *AtPK7*, and *AtPK8* are predominantly highly expressed in photosynthetically active tissues (e.g., leaves), with significant transcriptional signals also detected in developing inflorescences and young shoot apices, suggesting these subtypes are primarily involved in carbon flux partitioning and energy supply in photosynthetic tissues. *AtPK1* and *AtPK2* show higher expression in roots. Moreover, PKc expression is regulated by carbon source supply, under sufficient glucose conditions, multiple *PKc* genes are significantly induced in roots and cotyledons [27]. In rice, comprehensive expression profiling of 6 *OsPKc* genes was performed [18]. *OsPK3/5/10* are also expressed in panicles, and *OsPK3/10* are additionally expressed in roots [18]. *OsPK5* exhibits constitutive expression, with high levels in germinating seeds [35]. *OsPK3* is highly expressed in endosperm during the grain-filling stage [28]. *OsPK1* is broadly expressed across multiple tissues but is particularly prominent in stem nodes and panicle axes [29]. In tiger nut, *CePKc* genes exhibit markedly different expression patterns between tuber germination and seedling establishment stages: *CePKc1* and *CePKc2* are highly expressed in germinating tubers to provide energy for tuber sprouting, whereas *CePKc3* and *CePKc4* show elevated expression in seedling leaves, participating in carbon metabolism of photosynthetic tissues [26]. In soybean, *GmPK* family members exhibit pronounced tissue preferences, with some members highly expressed during seed development, associated with seed storage compound accumulation [21]. In *litchi*, *LcPK* expression levels are closely correlated with fruit development stages and postharvest physiological processes, with specific *LcPK* genes showing significant expression changes during fruit ripening and postharvest browning [23]. In peanut, *AhPK2/5/11/13/15* are highly expressed in pericarps, participating in pod development and hard shell formation, *AhPK19/21* are preferentially expressed in roots, mediating root growth and water absorption [22]. In developing oilseeds (such as castor beans and rapeseeds), PKc is highly expressed in the endosperm or cotyledons [40,56]; In loquat, *EjPKc* is highly expressed during fruit ripening, with markedly higher expression levels in high-sugar varieties compared to low-sugar ones, implying a potential role for *EjPKc* in regulating sugar content in loquat fruit [52]. In honeysuckle, the LjPkc gene exhibits broad expression in various tissues, and its transcript level is most abundant in yellow flowers [53]. The *DoPK* gene in *Dendrobium officinale* is constitutively expressed, and its relative expression level is highest in stems, moderate in roots, and lowest in leaves [54].

Overall, the tissue-specific expression patterns of PKc subtypes are highly consistent with the metabolic demands of their respective tissues. PKc is highly expressed in tissues with high energy requirements (e.g., developing seeds, actively growing shoot apices) and shows low expression in metabolically quiescent tissues. This refined expression regulation ensures precise matching of glycolytic flux with tissue metabolic demands.

#### 1.1.3. Subcellular Localization

The traditional view holds that PKc is exclusively localized in the cytoplasm, performing canonical glycolytic functions. Cytoplasmic localization represents the fundamental localization pattern of PKc. In the cytoplasm, PKc catalyzes the terminal step of glycolysis, providing ATP and pyruvate for the cell, and serves as a core enzyme maintaining basal respiratory metabolism [2,13].

However, recent studies have revealed dynamism and diversity in the subcellular localization of plant PKc, with localization status closely related to functional properties. Nuclear localization represents a newly identified functional localization of PKc. In *Arabidopsis*, AtPK6, AtPK7, and AtPK8 are primarily localized in the nucleus under conditions of sufficient glucose or light, whereas they migrate to the cytoplasm under sugar starvation or dark conditions, and re-enter the nucleus upon resumed sugar supply or illumination (Figure 1) [36]. In rice, OsPK1 is predominantly localized in the cytoplasm under normal temperatures but undergoes rapid nuclear translocation under heat stress (Figure 1) [30]. Notably, PKM2 in animal cells has also been reported to exhibit similar nuclear localization [57], suggesting evolutionary conservation of this non-canonical function.

Rice OsPK3 can form heteromeric complexes with OsPK1 or OsPK4, localizing to the outer mitochondrial membrane surface (Figure 1). This mitochondria-associated PK complex constructs a metabolic channel between the cytoplasm and mitochondria, enabling direct mitochondrial uptake of pyruvate produced by glycolysis, thereby maximizing respiratory metabolic efficiency during grain filling to support grain development [28]. Furthermore, a PK isozyme localized within a mitochondrial subcompartment (intrinsic mitochondrial PK) has been identified in Jerusalem artichoke (*Helianthus tuberosus*) tubers. Its pH sensitivity and inhibitor kinetics differ significantly from cytoplasmic PKc, possibly representing an independently evolved mitochondrial PK subtype [46].

The spatiotemporal regulation of PKc subcellular localization indicates that its functions extend far beyond glycolytic ATP production, integrating metabolic signals and gene expression regulation through shuttling between different subcellular compartments, thereby achieving multi-level regulation of plant growth and development.

#### 1.1.4. Protein–Protein Interaction Regulatory Networks

PKc function is finely regulated by a complex protein–protein interaction network, encompassing subunit–subunit interactions, upstream kinase regulation, and synergistic actions of stress-responsive proteins. Subunit–subunit interactions form the structural basis for PKc functional diversity. In rice, OsPK3 forms heteromeric complexes with OsPK1 or OsPK4, respectively (Figure 1). In potato, the plant energy sensor SnRK1 (SNF1-related protein kinase 1) directly physically interacts with PKc. Two isoforms of SnRK1, PKIN1 and StubSNF1, achieve this binding by recognizing specific motifs at the C-terminus of the PKc protein, with StubSNF1 playing a more prominent role in this regulatory process. As an “energy sensor” within plant cells, SnRK1 is activated when the AMP/ATP ratio increases (i.e., under energy-deficient conditions), and through its direct interaction with PKc, it rapidly modulates glycolytic flux, thereby maintaining the dynamic balance of energy metabolism [39]. Under saline-alkali stress, rice stress-associated protein OsSAP6 specifically binds to OsPK3 [34], collaboratively enhancing plant reactive oxygen species (ROS) scavenging capacity and improving tolerance to saline-alkali stress by stabilizing enzyme conformation or altering its subcellular distribution (Figure 1). Under heat stress, rice OsPK1-GCN5 forms a functional complex in the nucleus, and activation of this complex significantly upregulates the heat-responsive gene network including *HsfA2*, *Hsp70*, and *Hsp90*, improving the survival rate of transgenic rice under heat treatment by over 40% (Figure 1) [30]. In *Arabidopsis*, nuclear-localized AtPK6, AtPK7, and AtPK8 interact with the chromatin remodeling complex subunit SWC4, regulating transcription of flowering-related genes (Figure 1) [36]. This discovery extends PKc function from a metabolic enzyme to an epigenetic regulator, providing a novel perspective for understanding the coordination mechanism between plant development and metabolism.

#### 1.1.5. Activity Regulation

PKc exhibits complex allosteric regulatory characteristics, with its activity finely modulated by various metabolites, enabling PKc to sense and respond to changes in cellular metabolic status in real time [2,13].

Substrates and cofactors regulate PKc activity. PKc catalysis requires divalent metal ions (Mg^2+^ or Mn^2+^) and monovalent cations (K^+^) as cofactors, which are essential for maintaining the active conformation of the enzyme [2]. Substrate PEP and ADP concentrations directly affect reaction rates, and within the physiological concentration range, PKc affinity for PEP is significantly modulated by allosteric effectors [2]. Fructose-1,6-bisphosphate (FBP) is one of the most important positive allosteric activators of plant PKc. Elevated FBP concentrations signal increased upstream glycolytic flux, and PKc activation accelerates pyruvate production through a feed-forward activation mechanism [2,13]. Additionally, AMP can serve as a positive effector, activating PKc during energy deficit to accelerate ATP generation. ATP and alanine are the primary negative allosteric inhibitors of plant PKc. Elevated ATP concentrations (energy sufficiency) inhibit PKc activity, reducing unnecessary glycolytic consumption. Alanine, as the aminotransamination product of pyruvate, prevents excessive pyruvate accumulation through feedback inhibition of PKc [2,3]. Different species exhibit varying sensitivities of PKc to alanine: castor bean leaf PKc is highly sensitive to alanine inhibition, whereas banana fruit PKc is relatively insensitive to alanine [3,6].

Different PKc alleles also exhibit significant differences at allosteric regulatory sites, which directly affect their sensitivities to effectors and catalytic efficiencies. For instance, natural variation in the PKc protein encoded by rice *OsPK5* can lead to altered enzyme activity, thereby affecting glycolytic flux and seed germination efficiency [35].

In recent years, post-translational modifications (PTMs) have been found to regulate PKc activity. Phosphorylation typically inhibits PKc activity and promotes its degradation, whereas lysine acetylation can enhance enzyme activity. Soybean PKc harbors conserved phosphorylation sites, and its phosphorylation status affects both catalytic activity and protein stability [58]. During cotton fiber development, GhPK6 undergoes serine phosphorylation, which not only directly inhibits enzyme activity but also serves as a degradation signal that recruits the ubiquitination machinery, promoting GhPK6 degradation via the 26S proteasome pathway [24,59], thereby regulating cell elongation by reducing metabolic flux and ROS levels. Upon interaction with SWC4, *Arabidopsis* AtPK6, AtPK7, and AtPK8 directly participate in histone modification and transcriptional regulation, controlling the transcription of flowering-related genes by phosphorylating histone H3 at threonine 11 (H3T11ph) [36]. OsPK1 undergoes lysine acetylation under heat stress, which not only enhances its own enzymatic activity but also promotes histone H3K9 acetylation and H3T11 phosphorylation, thereby activating heat-responsive gene expression [30]. More remarkably, OsPK1 phosphorylates the histone acetyltransferase GCN5, which in turn acetylates OsPK1, forming a positive feedback loop that reinforces plant thermotolerance [30]. This discovery reveals a bidirectional interaction mechanism between metabolic enzymes and epigenetic regulators, and nuclear protein interactions further expand the functional boundaries of PKc. These modifications provide cells with rapid, reversible molecular means to regulate PKc activity, enabling swift responses to environmental changes without alterations in gene expression.

#### 1.1.6. Effects on Plant Growth and Development

As a central hub of energy metabolism, the functional status of PKc profoundly influences plant growth and development throughout the entire life cycle, encompassing vegetative growth, reproductive development, fruit quality, and stress adaptation.

PKc dysfunction significantly affects plant vegetative growth and morphology. The T-DNA insertion mutant of rice *OsPK1* exhibits a typical dwarf phenotype with shortened apical internodes, resulting in panicle enclosure [29]. On one hand, PKc deficiency leads to insufficient carbon skeleton supply, inhibiting parenchyma cell elongation and depriving cell wall synthesis of substrates; on the other hand, *OsPK1* silencing disrupts carbon metabolism, upregulating ABA biosynthesis genes and suppressing active GA biosynthesis, creating a hormonal imbalance characterized by “elevated ABA/reduced GA” [31]; The deficiency of PKc in tobacco leaves disturbs the source-sink relationship and impairs normal growth. Under low light, transgenic tobacco lines lacking leaf PKc show delayed stem and flower development and obvious leaf concavity [45].

PKc affects crop grain development. The rice *OsPK3* mutant exhibits reduced grain-filling rate and chalky grains, which is attributable not only to insufficient carbon skeleton supply affecting starch synthesis but also to source-sink transport impairment caused by dysregulated expression of sucrose transporters (*OsSUTs*, *OsSWEETs*), which is critical for grain filling [28].

PKc plays a critical role in seed germination. In rice, natural variation in *OsPK5* affects seed germination efficiency, and *OsPK5* loss-of-function leads to glucose accumulation, which blocks germination by inhibiting GA signaling and enhancing ABA signaling (upregulating *OsABI5*) [35]. *AhPK5/11/15/19/21* are significantly upregulated at 8 h after imbibition, suggesting their positive regulation of germination initiation, providing ATP for energy supply and metabolic reconstruction [22].

PKc also affects fruit quality. In persimmon (*Diospyros kaki*, Chinese pollination constant and non-astringency persimmon (C-PCNA) type), cytoplasmic PKc genes (*DkPK1*, *DkPK7*, *DkPK8*) play decisive roles in natural fruit deastringency [47,48,49]. These genes are highly expressed during late fruit development, and by accelerating glycolysis to produce abundant pyruvate, they upregulate the expression of downstream pyruvate decarboxylase (*DkPDC*) and alcohol dehydrogenase (*DkADH*), thereby achieving fruit deastringency [50,51]. In *litchi* fruit, PKc expression levels are closely correlated with respiratory climacteric and postharvest browning [23]. During cotton fiber development, *GhPK6* expression levels are negatively correlated with fiber elongation; moderate reduction in its activity decreases ROS accumulation, which in turn promotes fiber elongation [24].

PKc also participates in plant stress responses. Rice OsPK3 and OsSAP6 synergistically and significantly enhance tolerance to saline-alkali stress [34]. Rice OsPK1 strengthens thermotolerance through an acetylation-phosphorylation positive feedback loop [30]. RNAi silencing of potato *StPKcYT1* leads to pyruvate deficiency, which directly inhibits alternative oxidase (AOX) activity in vivo [19], weakening the mitochondrial buffering capacity against oxidative stress and rendering tissues more susceptible to programmed cell death. In peanut, the 2 kb upstream regions of *AhPKs* harbor abundant regulatory elements, including hormone-responsive, stress-responsive, light-responsive, and development-related elements, indicating that *AhPKs* are co-regulated by multiple environmental and endogenous signals [22]. The expression of pepper *CaPKc1* is induced by salicylic acid (SA), ethylene, and methyl jasmonate (MeJA), suggesting that this gene may be involved in viral defense signal transduction pathways that are dependent on SA/MeJA/ethylene [55].

### 1.2. Plastidic Pyruvate Kinase (PKp)

PKp was first isolated as an isozyme of pyruvate kinase from proplastids of developing castor bean endosperm [41]. Subsequently, pyruvate kinase activity was detected in both chloroplasts and etioplasts, and its biochemical properties were characterized [40]. The distinct roles of PKp and PKc with respect to subcellular localization, transcriptional regulation, and metabolic functionality underscore the regionalized organization and inherent complexity of plant cellular metabolism [60,61].

#### 1.2.1. Protein Size and Subunit Composition

PKp genes are typically fewer in number than PKc genes, but their protein structures are more complex, exhibiting unique heteromultimeric assembly characteristics. *Arabidopsis* contains 4 PKp genes (*AtPKp1*–*AtPKp4*), rice has 4 *OsPKp* genes [18], upland cotton contains 14 *GhPKp* genes [24], soybean harbors 4 *GmPKp* genes [20], tiger nut has 3 (*CePKpα*, *CePKpβ1*, *CePKpβ2*) [26], persimmon has 3 *DkPKp* genes [51], and peanut contains 10 PKp genes (5 *PKpα*, 5 *PKpβ*) [22].

PKp genes share a conserved PK catalytic domain with PKc but contain a unique plastid-targeting signal peptide sequence (Transit Peptide, TP). Due to the presence of the signal peptide, the precursor molecular weight of PKp proteins is slightly larger than that of PKc, typically 65–70 kDa, and the mature protein after signal peptide cleavage is approximately 55–60 kDa [2,13]. PKp from the green alga *Selenastrum minutum* was confirmed to be a homotetramer composed of a single ~56 kDa subunit [20].

Sequence homology analysis reveals that plant PKp genes can be classified into two subfamilies, α and β, which exhibit significant sequence divergence (typically only 30–40% homology) but must work cooperatively to form a functional enzyme complex [37,38]. This divergence represents a unique characteristic of plant plastidic PK and has not been observed in bacteria or animals [11,12]. The PKp-α subunit (also designated PKp1), although possessing independent catalytic activity, exhibits very low and unstable activity; the PKp-β subunit (also designated PKp2) has almost no independent catalytic activity and primarily serves a regulatory function. Notably, the β1 and β2 subtypes differ in both sequence and function. In *Arabidopsis*, α-β1 complexes and α-β2 complexes differ in catalytic efficiency, allosteric regulatory sensitivity, and tissue expression patterns [37,38].

#### 1.2.2. Tissue- and Organ-Specific Expression Profiles

PKp expression patterns are strictly regulated by tissue-specific promoters, highly consistent with the tissue distribution of its associated metabolic pathways. In photosynthetic tissues, PKp participates in the glycolytic pathway within chloroplasts, providing carbon skeletons for photorespiration. PK activity in leaf chloroplasts is regulated by photosynthetic products and energy status under light conditions [1]. In non-photosynthetic tissues such as roots, stems, and developing seeds, PKp is involved in supplying precursors for starch synthesis, and jointly regulates starch biosynthesis together with ADP-glucose pyrophosphorylase (AGPase), starch synthases, and other enzymes [32,33].

In *Arabidopsis*, *AtPKpβ1* and *AtPKpβ2* are predominantly highly expressed in developing seeds, highly coinciding with the spatiotemporal pattern of seed oil accumulation [37,38]. *AtPKpβ1* expression peaks during mid-seed development (the rapid oil accumulation stage) and subsequently declines during seed maturation, which aligns with the dynamic changes in pyruvate demand for fatty acid synthesis [38]. In rice, *OsPK2* (encoding PKpα1) is specifically highly expressed during endosperm development, with the highest expression levels during the mid-grain-filling stage (the rapid starch accumulation stage) [32,33]. The OsPKpα1 subunit is specifically localized in the stroma of seed amyloplasts, co-localizing with starch synthesis enzymes in the same subcellular compartment, thereby establishing the spatial foundation for its direct utilization of ADP produced from ADP-glucose pyrophosphorylase reactions and for supplying pyruvate backbones. In upland cotton, PKp genes (*GhPK1*, *GhPK5*, *GhPK27*, *GhPK28*) are preferentially expressed in ovules and developing fiber cells, coinciding with the rapid accumulation period of oil and protein in cottonseeds [24]. In peanut, *AhPKp6/16* genes are highly expressed during seed development and are strongly induced by ABA and drought stress [22]. Oilseed rape PKp is also highly expressed in seeds, participating in fatty acid synthesis in oilseed crop seeds [62].

#### 1.2.3. Subcellular Localization

PKp localization is determined by its N-terminal transit peptide, which directs the nascent protein into the plastid stroma. PKp is produced as a precursor in the cytosol, featuring a plastid transit peptide at its N-terminus that facilitates its import across the plastid envelope into the stroma, where the transit peptide is removed by processing proteases to generate the active enzyme [9,24]. PKc, however, does not possess a transit peptide and becomes functional immediately upon synthesis in the cytosol [63].

PKp is present in various types of plastids. In green photosynthetic tissues, it is localized in chloroplasts. In etiolated seedlings, it is localized in etioplasts, indicating its important functions in non-photosynthetic plastids [40]. In developing castor bean endosperm, it is localized in leucoplasts, where it supplies carbon skeletons for fatty acid synthesis [41,42,43]. In rice endosperm, it is localized in amyloplasts and is involved in metabolism related to starch synthesis [32,33].

#### 1.2.4. Protein–Protein Interaction Regulatory Networks

Compared to the established body of research on PKc, research on PKp protein–protein interactions is relatively limited. However, it is known that PKp primarily forms active complexes through subunit–subunit interactions and is subject to multidimensional regulation by metabolite feedback, hormonal signals, and environmental factors [32,33,37,38]. In *Arabidopsis*, functional PKp complexes have been confirmed to be heterooctamers (hypothesized to be a 4α:4β configuration) assembled from catalytic α subunits and regulatory β subunits [37,38]. This heteromultimeric assembly strategy enables PKp to separate catalytic from regulatory functions, adapting to different tissue metabolic demands by altering subunit composition. In rice, the PKp complex is cooperatively composed of α family subunits (OsPKpα1/OsPK2, OsPKpα2) and β family subunits (OsPKpβ1, OsPKpβ2). Functional studies of *OsPK2* (encoding the PKpα1 subunit) demonstrate that the α subunit is the core of PKp complex catalytic activity, and its absence leads to a substantial decrease in total PKp activity [32,33]. Peanut PKp subfamily members likewise function through heteromultimeric assembly of α and β subunits [22]. Castor PKp functions as a single hybrid enzyme co-encoded by the PKpA and PKpG genes, which naturally assembles into an α_2_β_4_ heterohexameric structure (consisting of two α and four β subunits) [44].

#### 1.2.5. Activity Regulation

The activity regulation mechanisms of PKp share commonalities with PKc but also exhibit unique plastid-specific characteristics. Metabolite feedback regulation within plastids: PKp activity is finely modulated by various plastidial metabolites. Glutamate (Glu), as a central node of nitrogen metabolism, strongly allosterically inhibits the *Arabidopsis* α-β1 complex, whereas the α-β2 complex is more sensitive to regulation by oxaloacetate (OAA) and exhibits distinct response characteristics to fructose-6-phosphate (F6P) [37,38]. The ATP/ADP ratio also affects PKp activity; however, because plastidial ATP is primarily derived from photophosphorylation, its regulatory mechanism differs from that in the cytoplasm.

PKp activity is also pH-dependent: PKp typically exhibits an alkaline pH optimum (approximately pH 8.0), which is highly consistent with the physiological change in chloroplast stroma alkalinization during illumination (pH rises from approximately 7.0 to approximately 8.0) [2,13].

Also, PKp functional stability is strictly regulated by inter-subunit dependencies. In *Arabidopsis*, the α-β1 and α-β2 complexes exhibit significant differences in kinetic parameters (Km, Vmax) and allosteric regulatory sensitivity. Loss-of-function of the β1 subunit gene leads to a substantial reduction in total PKp activity, which cannot be restored by overexpressing the α subunit [38], indicating that the β1 subunit is indispensable for maintaining the stability and active conformation of the α subunit. In rice, the α1 subunit can not only form heteromeric complexes with β subunits but also exhibits monomeric self-interaction capability; however, the catalytic activity of the monomeric form is far lower than that of the heteromeric complex [32,33].

PKp gene transcription is directly regulated by plant hormones such as abscisic acid (ABA). The promoter regions of peanut *AhPKp* genes are enriched in ABRE elements (ABA-responsive element), and expression profile analysis confirmed that these genes are strongly induced by exogenous ABA and PEG (polyethylene glycol) -simulated drought stress [22].

Significant impacts of environmental stress on PKp protein stability and activity have been observed. In soybean subjected to waterlogging stress, PKp in hypocotyls is significantly upregulated during the anaerobic hypoxia stage, with increased protein abundance, to maintain anaerobic glycolytic flux. However, during the reoxygenation recovery phase, its mRNA level, protein abundance, and enzymatic activity all rapidly return to normal levels [20]. In upland cotton, *GhPKp* genes exhibit complex interactive responses to heat and drought stress: under single heat or drought stress, *GhPKp* expression tends to be downregulated, which may represent a metabolic suppression strategy to reduce energy consumption. However, under combined heat and drought stress, *GhPKp* expression is significantly upregulated [25].

#### 1.2.6. Effects on Plant Growth and Development

PKp directly determines the partitioning ratio of carbon flux within plastids toward fatty acid, starch, or secondary metabolic pathways, profoundly influencing crop seed quality, yield formation, and stress adaptation capacity.

In starch crops such as rice, PKp dysfunction induces unique quality deterioration. The low-expression mutant of *OsPK2* (encoding PKpα1) exhibits a typical floury endosperm phenotype and impaired grain filling. Ultrastructural observations revealed that amyloplast compound starch granule formation was disrupted in the mutant, with starch granules displaying small size and irregular dispersion, indicating that *OsPK2* is crucial for starch synthase activity and normal starch granule assembly. Notably, the chalky mutant caused by *OsPK2* deficiency is also accompanied by a reduced number of oil bodies in the aleurone layer and downregulated expression of fatty acid synthesis genes, suggesting that in rice endosperm, PKp simultaneously co-regulates both starch and fatty acid metabolic branches by providing the common substrate (pyruvate). *OsPK2* deficiency leads to a comprehensive decrease in fatty acid content, including fatty acids with chain lengths of 14, 16, and 18–24 carbons thereby affecting cell membrane lipid synthesis and grain filling [32,33]. In oilseed crops, PKp serves as the primary carbon source supplier for fatty acid biosynthesis.

In *Arabidopsis*, loss-of-function of the regulatory β1 subunit leads to a substantial decrease in total PKp activity, directly resulting in approximately 60% reduction in mature seed oil content and shrunken seed morphology. Only complementation with the β1 subunit can fully restore oil content, indicating that β1-mediated PKp holoenzyme assembly is the rate-limiting step for oil accumulation [38]. This finding provides an important target for improving oil content in oilseed crops through genetic manipulation of PKp.

Also, PKp plays a crucial role in mobilizing nutrients required for seed germination. The *Arabidopsis* PKp mutant cannot effectively utilize its own stored lipids during germination (despite already reduced oil content) and must rely on exogenous sucrose supply to complete seedling establishment [64]. In peanut, PKp genes such as *AhPK5* and *AhPK11* are explosively expressed in embryos of imbibed seeds, representing a necessary condition for initiating the germination program [22]. This indicates that PKp not only participates in lipid accumulation during seed development but also supports the mobilization and utilization of stored lipids during germination by providing pyruvate.

## 2. Analysis Method

### 2.1. Phylogenetic Tree Construction and Synteny Analysis

The full-length protein sequences of pyruvate kinase (PK) family members from six crop species were retrieved from public databases (Table S1) and subjected to phylogenetic analysis. A phylogenetic tree was constructed using the Maximum Likelihood (ML) method in MEGA 12 software (Version 12.0), with 1000 bootstrap replicates to evaluate the reliability of each clade. Meanwhile, conserved motifs were identified using the MEME online tool (https://meme-suite.org/meme/, accessed on 12 December 2025), with the maximum number of motifs set to 10, and conserved domain annotations were performed using the NCBI Conserved Domain Database (CDD, https://www.ncbi.nlm.nih.gov/cdd, accessed on 15 December 2025). The phylogenetic tree, motif distribution, and domain architecture were integrated and visualized using the ChiPlot online platform (https://www.chiplot.online/, accessed on 5 January 2026) (Figure 2, Tables S1–S3) [65,66].

To further investigate the genomic-level evolutionary patterns of the PK gene family beyond the phylogenetic analysis, synteny analysis was conducted using the model plants rice (*Oryza sativa*) and *Arabidopsis thaliana* and visualized using TBtools II software (Version v2.485) (Figure 3).

### 2.2. Cis-Acting Element Analysis

The genomic sequences and corresponding annotation files of six plant species were retrieved from public databases. For each gene, a 2000 bp sequence upstream of the transcription start site was extracted as the putative promoter region. Cis-acting elements were predicted using the PlantCARE online tool (https://bioinformatics.psb.ugent.be/webtools/plantcare/html/, accessed on 14 January 2026). Based on previously published studies on plant promoter cis-elements [67,68,69], a total of 25 representative cis-elements with well-characterized functions were selected from the prediction results, covering core promoter/basal transcription, light responsiveness, plant hormone responsiveness, and biotic/abiotic stress responsiveness (Figure 4 and Figure S1, Tables S4 and S5). The results were visualized using TBtools II software [70].

## 3. Conclusions

PKc and PKp, as key enzymes of the glycolytic pathway, form a distinct division of labor and coordination in gene origin, protein structure, subcellular localization, and metabolic functions. PKc originated from the nuclear genome of the eukaryotic host and is widely distributed in the cytosol, nucleus, and around mitochondria, primarily functioning in basal respiratory energy supply, epigenetic regulation, and integration of stress signals. PKp originated from endosymbiotic cyanobacteria, is exclusively localized in plastids, and drives fatty acid, starch, and secondary metabolism (Table 2) [71]. Through refined expression regulation, diverse subunit combination patterns, and post-translational modifications, they collectively achieve complex regulatory network of the plant carbon metabolic network.

This review summarizes the expansion of plant PKc function from the traditional role as the “terminal rate-limiting enzyme of glycolysis” to a “metabolic–epigenetic cross-domain regulator,” systematically compares the diversity of PKp heteromultimeric structures across species such as castor bean, Arabidopsis, and rice, and integrates the evolutionary data of PK gene families and promoter cis-element analyses from six plant species with extensive PK research, thereby providing a theoretical foundation for PK-based molecular breeding in crops.

Current research has revealed the basic characteristics and partial functions of PKc and PKp; however, numerous scientific questions remain to be addressed. Firstly, the intracellular trafficking of PKc, specifically its transit between the nucleus and mitochondria remains a biological black box. By investigating how PKc senses energy status and whether its NLS is fine-tuned by post-translational modifications, will deepen our understanding of metabolism–epigenetics crosstalk. Secondly, the interaction networks between PKp and other metabolic pathways within plastids warrant systematic elucidation. How does PKp coordinate and interact with the fatty acid synthase system, starch synthase system, and MEP pathway? Integrated analysis of plastid metabolomics and proteomics will provide novel clues for this endeavor. Lastly, the evolutionary patterns and functional diversification mechanisms of the PK family across different species require in-depth investigation. Have the extensively expanded PK genes in polyploid crops (e.g., soybean, cotton, peanut) undergone functional diversification? Integration of comparative genomics with functional validation will serve as an effective approach to address this question.

Based on the critical roles of PK subtypes in seed vigor, stress response, and yield formation, they have emerged as candidate targets for crop genetic improvement. Given that superior haplotypes have already been identified in this gene family [35], future research can combine multi-omics analysis with genome-wide association studies (GWAS) to systematically exploit superior allelic variations within PK subtypes, especially superior haplotypes under various stress conditions, thereby accelerating the breeding application of this gene family under the current persistently unfavorable climatic conditions.

## Figures and Tables

**Table 1:** Subcellular localization and function of PK genes/proteins in various plant species.

Plant Species	Gene/Protein	Subcellular Localization	Main Function	References
Rice (*Oryza sativa*)	OsPK1 (PKc)	Cytoplasm (normal); Nucleus (heat stress)/Mitochondrial outer membrane	Regulates plant height, heat stress response; nuclear interaction with GCN5 activates heat-responsive genes	[28,29,30,31]
	OsPK2 (PKpα1)	Amyloplast stroma	Endosperm starch synthesis, compound starch granule formation, grain filling	[32,33]
	OsPK3 (PKc)	Cytoplasm/Mitochondrial outer membrane	Grain filling; interaction with OsSAP6 enhances salt-alkali tolerance	[28,34]
	OsPK4 (PKc)	Cytoplasm/Mitochondrial outer membrane	Forms heteromeric complex with OsPK3	[18,28]
	OsPK5 (PKc)	Cytoplasm	Seed germination regulation (natural variation affects germination efficiency)	[35]
	OsPK7, OsPK8, OsPK9, OsPK10	Cytoplasm	Differential tissue expression, involved in carbon metabolism	[18]
Arabidopsis (*Arabidopsis thaliana*)	AtPK1–10 (PKc)	Cytoplasm; AtPK6/7/8 can enter nucleus	Energy metabolism; AtPK6/7/8 regulate flowering gene transcription	[36]
	AtPKp1–4 (PKp)	Plastid (AtPKpβ1/β2 highly expressed in developing seeds)	Seed oil accumulation (AtPKpβ1 loss of function → 60% reduction in oil)	[37,38]
Soybean (*Glycine max*)	GmPK1–27 (16 PKc, 4 PKp)	Cytoplasm (PKc); Plastid (PKp)	Seed development; PKp upregulation maintains anaerobic glycolysis under waterlogging	[21]
Peanut (*Arachis hypogaea*)	AhPK1–21 (11 PKc, 10 PKp)	Cytoplasm (PKc); Plastid (PKp)	AhPK2/5/11/13/15 highly expressed in fruits; AhPK19/21 expressed in roots; seed germination	[22]
Potato (*Solanum tuberosum*)	StPKcYT1–5 (PKc)	Cytoplasm	Interacts with SnRK1 to regulate glycolytic flux; RNAi silencing → pyruvate deficiency → AOX activity inhibition	[19,39]
Upland Cotton (*Gossypium hirsutum*)	GhPK1–33 (19 PKc, 14 PKp)	Cytoplasm (PKc); Plastid (PKp)	GhPK6 expression negatively correlated with fiber elongation; PKp highly expressed in ovules and fiber cells	[24,25]
Castor Bean (*Ricinus communis*)	PKc (~57 kDa + ~56 kDa subunits)	Cytoplasm	Energy supply for developing embryos, provides carbon for fatty acid synthesis	[3,40]
	PKp (α_2_β_4_ heterooctamer)	Leucoplast	Provides carbon skeleton for fatty acid synthesis	[41,42,43,44]
Tobacco (*Nicotiana tabacum*)	PKc (heterotetramer)	Cytoplasm	Leaf carbon distribution; PKc deficiency disrupts source–sink relationship	[3,45]
	PKp	Chloroplast	Photosynthetic tissue plastid glycolysis	[9]
Banana (*Musa acuminata*)	PKc (homotetramer, ~57 kDa subunit)	Cytoplasm	Fruit glycolysis, fruit ripening	[6]
Green Alga (*Selenastrum minutum*)	PKc (homotetramer, 57 kDa)	Cytoplasm	Lower plant glycolysis	[20]
	PKp (homotetramer, ~56 kDa)	Plastid	Plastid glycolysis	[20]
Jerusalem Artichoke (*Helianthus tuberosus*)	Mitochondrial PK (mitochondrial PK isozyme)	Mitochondrial subcompartment	Mitochondrial pyruvate metabolism, distinct biochemical properties from cytoplasmic PKc	[46]
Persimmon (*Diospyros kaki*, C-PCNA type)	DkPK1, DkPK7, DkPK8 (PKc)	Cytoplasm	Natural fruit de-astringency (accelerated glycolysis → pyruvate → upregulation of DkPDC/DkADH)	[47,48,49,50,51]
	DkPKp1–3 (PKp)	Plastid	Plastid metabolism	[51]
*Litchi* (*Litchi chinensis*)	LcPK1–19	Cytoplasm/Plastid	Fruit development, post-harvest browning related to respiratory burst	[23]
Tiger Nut (*Cyperus esculentus*)	CePKc1–4 (PKc); CePKpα, β1, β2 (PKp)	Cytoplasm (PKc); Plastid (PKp)	CePKc1/2 highly expressed during tuber sprouting; CePKc3/4 highly expressed in seedling leaves	[26]
Loquat (*Eriobotrya japonica*)	EjPKc	Cytoplasm	Fruit ripening, higher expression in high-sugar varieties → regulates fruit sugar content	[52]
Honeysuckle (*Lonicera japonica*)	LjPKc	Cytoplasm (widely expressed, highest in flowers)	Multi-tissue metabolism	[53]
Dendrobium (*Dendrobium officinale*)	DoPK	Cytoplasm (constitutively expressed, highest in stems)	Energy metabolism in different tissues	[54]
Pepper (*Capsicum annuum*)	CaPKc1	Cytoplasm	Induced by SA, ethylene, MeJA → may participate in virus defense signal transduction	[55]

**Table 2:** Functional comparison between PKc and PKp.

Feature	Cytosolic PK (PKc)	Plastidic PK (PKp)
Evolutionary origin	Eukaryotic host nuclear genome	Cyanobacterial endosymbiosis; prokaryotic gene transferred to plant nuclear genome
Sequence similarity	>50% among PKc (similar to mammalian PK)	Only 35–40% between PKc and PKp; 30–40% between PKp-α and PKp-β
Protein size	Mature protein ~55–60 kDa (500–530 aa)	Precursor 65–70 kDa; mature protein 55–60 kDa (including transit peptide)
Subcellular localization	Cytoplasm; dynamic distribution to nucleus and mitochondria	Various plastids (chloroplast, leucoplast, amyloplast, etioplast)
Assembly form	Homo/hetero-polymer (tetramer dominant)	Heteromeric complex (α–β complex; *Arabidopsis* is 4α:4β hetero-octamer)
Cofactor requirement	Mg^2+^/Mn^2+^, K^+^	Same as PKc
Allosteric regulation	FBP (activation), ATP (inhibition), alanine (inhibition)	Glu (inhibits α–β1), OAA (regulates α–β2), F6P response
Optimal pH	~6.5–7.5 (cytoplasm)	~8.0 (consistent with light-induced chloroplast stroma alkalization)
Main function	Glycolytic energy production; nuclear transcriptional regulation; mitochondrial pyruvate supply	Plastid PEP → pyruvate; supply for fatty acid synthesis; starch synthesis precursor supply
Dynamic localization	Nucleocytoplasmic shuttling (glucose/light signals); can localize to mitochondrial outer membrane	None (stably localized in plastid stroma)
Post-translational modification	Phosphorylation (inhibition + degradation); acetylation (activation + nuclear import)	Subunit interaction maintains stability
Crop improvement target	Plant height (OsPK1), germination (OsPK5), heat tolerance (OsPK1)	Starch quality (OsPK2), oil content (AtPKpβ1)

## Data Availability

No new data were created or analyzed in this study. Data sharing is not applicable to this article.

## Supplementary Materials

The following supporting information can be downloaded at: https://www.mdpi.com/article/10.3390/ijms27146346/s1.

## Abbreviation

The following abbreviations are used in this manuscript:

ABA	Abscisic acid
AGPase	ADP-glucose pyrophosphorylase
AOX	Alternative oxidase
ATP	Adenosine triphosphate
DXP	1-deoxy-D-xylulose-5-phosphate
DXR	1-deoxy-D-xylulose-5-phosphate reductoisomerase
F6P	Fructose-6-phosphate
FBP	Fructose-1,6-bisphosphate
GA	Gibberellic acid
GCN5	General control non-repressed 5
GWAS	Genome-wide association study
H3K9ac	Histone H3 lysine 9 acetylation
H3T11ph	Histone H3 threonine 11 phosphorylation
HK	Hexokinase
MEP	Methylerythritol phosphate
MeJA	Methyl jasmonate
MEME	Multiple EM for Motif Elicitation
MPC	Mitochondrial pyruvate carrier
NCBI	National Center for Biotechnology Information
NLS	Nuclear localization signal
OAA	Oxaloacetate
OsSAP6	Oryza sativa stress-associated protein 6
PC	Pyruvate carboxylase
PDC	Pyruvate dehydrogenase complex
PEG	Polyethylene glycol
PEPC	Phosphoenolpyruvate carboxylase
PEP	Phosphoenolpyruvate
PFK	Phosphofructokinase
Pfam	Protein families database
PK	Pyruvate kinase
PKc	Cytosolic-type pyruvate kinase
PKp	Plastidic-type pyruvate kinase
PKpα	Plastidic pyruvate kinase alpha subunit
PKpβ	Plastidic pyruvate kinase beta subunit
PPT	Phosphoenolpyruvate/Phosphate Translocator
PlantCARE	Plant Cis-acting Regulatory Elements database
ROS	Reactive oxygen species
SA	Salicylic acid
SnRK1	SNF1-related protein kinase 1
SWC4	SWI2/SNF2-like chromatin remodeling complex subunit 4
TCA	Tricarboxylic acid
TP	Transit peptide
